# Association between the dietary index for gut microbiota and metabolic syndrome: the mediating role of the dietary inflammatory index

**DOI:** 10.3389/fnut.2025.1617287

**Published:** 2025-07-21

**Authors:** Yu-Nan Han, Yong-Xiang Wang, Cheng-Yue Xiong, Lin Li, Ru-Meng Mao

**Affiliations:** ^1^Department of Endocrinology, The First Affiliated Hospital of Yangtze University, Jingzhou, China; ^2^Department of Medicine, Yangtze University, Jingzhou, China

**Keywords:** metabolic syndrome, gut microbiota diet index, dietary inflammatory index, mediation analysis, NHANES

## Abstract

**Background:**

Metabolic syndrome (MetS) poses a huge global public health challenge. The dietary index for gut microbiota (DI-GM) measures the influence of diets on the microbiome, but its link with the odds of MetS is elusive. This paper examined the link between DI-GM and MetS and investigated the mediating role of the dietary inflammatory index (DII).

**Methods:**

Data were sourced from the 2007–2018 NHANES for adults diagnosed with MetS. A total of 20,999 participants were included in the analysis. Dietary data were recorded via two 24-h dietary recalls, from which DI-GM and DII were calculated. Multivariate weighted logistic regression and restricted cubic spline (RCS) analyses were leveraged to assess the link between DI-GM and MetS. Mediation analysis was implemented to determine the role of DII in this association. Subgroup and sensitivity analyses were also implemented.

**Results:**

After adjusting for all covariates, participants in the highest DI-GM scores (Q4) had a greatly lower odds of MetS compared to those in the lowest DI-GM group (Q1) (OR = 0.80, 95% CI = 0.69–0.92, *p* = 0.003). The RCS analysis noted a linear negative link between DI-GM and MetS (P for nonlinear = 0.414). DII partially mediated the correlation between DI-GM and MetS [Indirect effect estimate = −0.00265, 95% CI: (−0.00445, −0.00167), *p* < 0.001]. Subgroup analysis showed consistent negative associations between higher DI-GM (Q4) and MetS across various demographic and lifestyle subgroups, with no statistically significant interactions observed (P interaction > 0.05).

**Conclusion:**

High DI-GM levels are linked with a significantly reduced odds of MetS, with DII partially mediating this association.

## 1 Introduction

Metabolic syndrome (MetS) is marked by disturbances in lipid and carbohydrate metabolism. As National Cholesterol Education Program Adult Treatment Panel III (NCEP-ATP III) criteria propose, MetS is primarily characterized by central obesity, declined high-density lipoprotein cholesterol (HDL-C) levels, enhanced triglycerides (TG), hypertension, and hyperglycemia (1). Over recent decades, MetS prevalence has risen markedly. In the United States (U.S.), MetS prevalence rose from 25.29 to 34.7% between 1988 and 2016 (2). MetS has been recognized as a significant public health burden, with substantial evidence linking it to an enhanced odds of coronary heart disease, stroke, cardiovascular disease (CVD), cancer, type 2 diabetes mellitus (DM), and all-cause mortality (3–5). Given these associations, MetS has been identified as a major public health concern that requires focused attention and intervention.

The significance of gut microbiota in metabolic disorders has garnered much attention. Research has demonstrated that gut microbiota dysbiosis is crucial in MetS progression by influencing host immune function, intestinal barrier integrity, and metabolite synthesis (6, 7). Consequently, maintaining healthy gut microbiota is believed to be promising way for MetS prevention and management. Diet is crucial for the composition and property of gut microbiota, and dietary patterns greatly influence microbiota diversity and their metabolites, thereby impacting host metabolic health (8). A systematic review concluded that dietary index for gut microbiota (DI-GM) could appraise dietary quality related to the maintenance of healthy gut microbiota (9).

Diet has been recognized as a primary factor in regulating inflammatory responses. Research has noted that a higher dietary inflammatory index (DII) is greatly linked with an enhanced odds of MetS. Elevated DII scores are linked not only to a higher likelihood of MetS in both younger and older populations but also to its key components, including heightened waist circumference (WC), body mass index (BMI), TG, blood glucose levels, and reduced HDL-C. The anti-inflammatory properties of dietary patterns may make them pivotal intervention strategies for addressing MetS (10, 11). The DII evaluates the inflammatory potential of diets and quantifying the effects of various foods on systemic inflammation. A higher DII score is indicative of a pro-inflammatory diet, and a lower DII infers an anti-inflammatory diet (12). Nonetheless, the mediating role of DII in the link between DI-GM and MetS has not been thoroughly investigated. Given that MetS is characterized by multiple concurrent metabolic dysregulations, elucidating the mediating effects of DII in the DI-GM–MetS link is of considerable scientific and clinical significance.

To date, the link between DI-GM and MetS and the potential mediating role of DII have not yet been investigated. This paper was therefore designed to determine the link between DI-GM and MetS using NHANES data from 2007 to 2018. Additionally, the potential mediating effect of DII was assessed. The findings may contribute to evidence-based strategies for MetS management.

## 2 Materials and methods

### 2.1 Data source and participants

**Figure 1:**
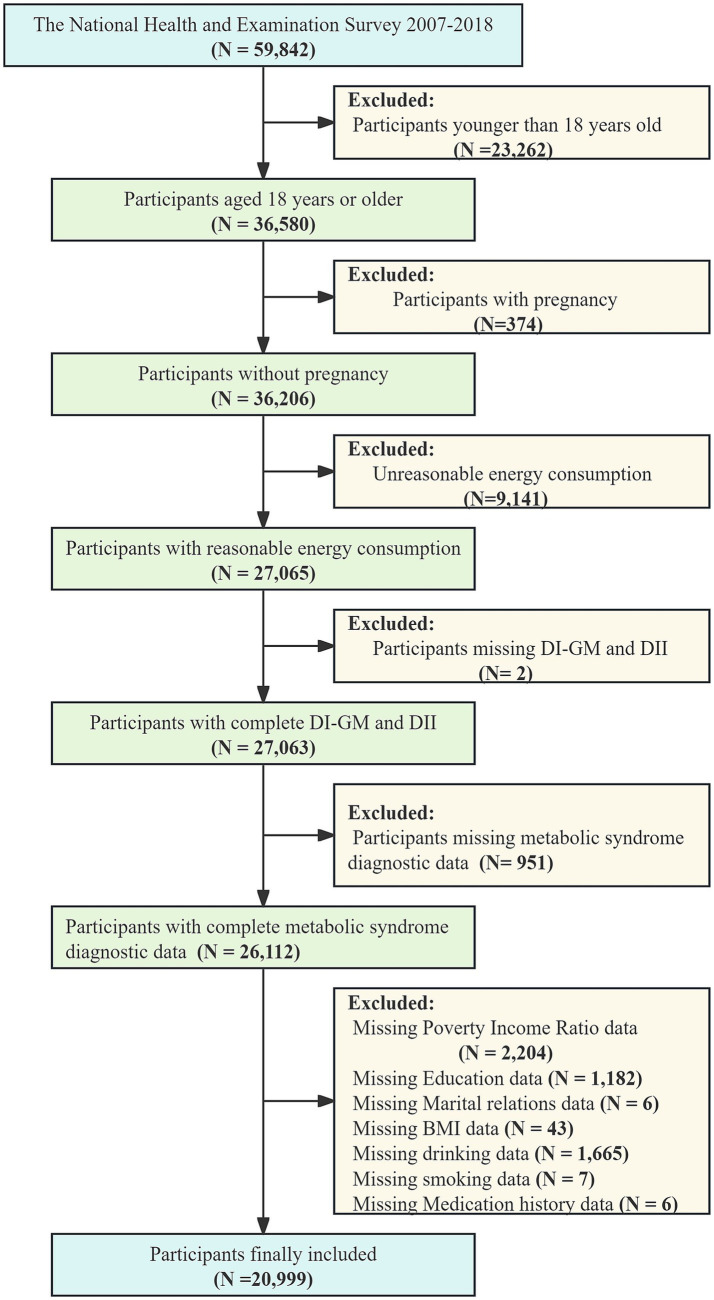
Flowchart of the selection strategy.

The NHANES is implemented by the Centers for Disease Control and Prevention and utilizes a complex, multistage probability sampling design. This survey aims to inspect the health and nutritional conditions of both U. S. adults and children. Data collection and release were carried out biennially, covering demographics, dietary patterns, examination parameters, laboratory data, and questionnaires. The NHANES protocol was ratified by the Research Ethics Review Board, and all participants offered written informed consent before their inclusion. For this cross-sectional analysis, data from six NHANES cycles from 2007 to 2018 were utilized. Participants were excluded for age under 18 years (*N* = 23, 262), pregnancy (*N* = 374), implausible energy intake (defined as <800 kcal/day or >5,000 kcal/day, *N* = 9,141), missing data for DI-GM and the mediating variable DII (*N* = 2), missing data for MetS (*N* = 951), and missing covariates (*N* = 5,113). Finally, 20, 999 participants were enrolled (Figure 1).

### 2.2 Assessment of MetS

MetS was diagnosed as per the criteria established by the NCEP-ATP III (13). The presence of three or more of the five following criteria was considered diagnostic of MetS. The diagnostic criteria included: (1) TG levels ≥ 1.69 mmol/L (150 mg/dL); (2) Reduced HDL-C: < 1.03 mmol/L (40 mg/dL) for men and < 1.29 mmol/L (50 mg/dL) for women; (3) Enhanced fasting plasma glucose (FPG) ≥ 6.1 mmol/L (110 mg/dL); (4) Increased WC: > 102 cm for men and > 88 cm for women; and (5) Systolic blood pressure (BP) ≥ 130 mmHg and/or diastolic BP ≥ 85 mmHg. Blood samples were collected in the morning following a 9-h fasting period. BP was measured twice by a physician to record the average value for analysis.

### 2.3 Assessment of DI-GM

DI-GM is designed to show the influence of dietary patterns on microbiota diversity and to present dietary features that promote beneficial gut microbiota (9). This novel index was developed through a systematic review of scientific literature, identifying 14 dietary components with beneficial or unfavorable effects on gut microbiota composition, diversity, and function (e.g., short-chain fatty acid production). Its construct validity was subsequently evaluated and confirmed in a large-scale population-based study, demonstrating its association with indirect biomarkers of gut microbiota diversity. Dietary data were collected via two 24-h dietary recalls, specifically the Automated Multiple-Pass Method (14). The standardized interview was conducted by trained interviewers, who recorded all foods and beverages participants had consumed over the past 24 h. This ensured data quality, thereby reducing interviewer bias and participant recall error (15). The average was used for analyses. DI-GM comprised 14 components, categorized into two groups: (1) 10 beneficial components: avocado, chickpeas, broccoli, coffee, cranberries, fermented dairy products, dietary fiber, soy, green tea (excluded in some analyses due to the absence of green tea consumption data in NHANES), and whole grains; and (2) 4 detrimental components: processed meat, red meat, refined grains, and a high-fat diet (fat energy ≥ 40% of total energy). For each component, the intake amount was quantified using the USDA Food and Nutrient Database for Dietary Studies (FNDDS) based on the 24-h recall data. The units of consumption varied by component, generally being grams per day (g/day) for most foods and nutrients (e.g., dietary fiber) or converted to servings per day (serving/day) for certain food groups as applicable from the NHANES data. For the high-fat diet component, the measure was the percentage of energy derived from fat. Based on sex-specific median level, beneficial components with intake over the median were scored 1 point, while those below were scored 0 point, with a range of 0 to 9 points; detrimental components with intake below the median were scored 1 point, while those above were scored 0 point (for the high-fat diet, a fat energy ratio < 40% scored 1 point), with a range of 0 to 4 points (Supplementary Table 1). Total DI-GM score ranged from 0 to 13. A higher DI-GM score indicated more potential dietary benefits for gut microbiota.

### 2.4 Assessment of DII

DII is an indicator adopted to appraise the potential inflammation of an individual’s diet. It involves 45 dietary components sourced from scientific literature, which are determined based on their effects on inflammatory indicators, involving C-reactive protein (CRP), interlrukin-6 (IL-6), and tumor necrosis factor-alpha (TNF-*α*). The scoring scale for each component ranges from −1 (anti-inflammatory) to +1 (pro-inflammatory) (16). The Food Frequency Questionnaire or 24-h dietary review was applied to assess participants’ dietary intake, and considering the differences in energy intake, the intake was standardized to per 1,000 kcal. Subsequently, the intake was adjusted using the formula [(daily intake - global average daily intake) divided by the standard deviation of the global average daily intake], and the modified intake was multiplied by the cumulative inflammatory response score of dietary components (17). The total DII score was computed, with a higher score implying a greater consumption of pro-inflammatory foods. The information on DII dietary components is detailed in Supplementary Table 2.

### 2.5 Assessment of covariates

Under prior research and clinical evaluations, a comprehensive set of potential confounding variables was examined, encompassing age, sex (male or female), race, marital status, education, poverty income ratio (PIR) (≤1.3, 1.3–3.5, and >3.5) (18, 19), smoking status (20), alcohol consumption (20), energy intake (a continuous variable) (21), and medication history (a binary (yes/no) variable) (22). Macronutrient intake, specifically protein, total fat, and carbohydrates, was also quantified from the two 24-h dietary recalls. Additionally, physical activity levels were categorized based on current guidelines for adults: meeting recommendations (> = 600 MET-minutes/week, equivalent to at least 150 min/week of moderate-intensity or 75 min/week of vigorous-intensity activity) or not meeting recommendations (<600 MET-minutes/week) (23). Smoking status involved never smokers (lifetime consumption of < 100 cigarettes), former smokers (≥100 cigarettes but currently quit), and current smokers (≥100 cigarettes and currently smoking occasionally or daily) (24). Alcohol consumption was divided into three groups: never drinkers (lifetime consumption of < 12 drinks), former drinkers (consumed ≥12 drinks in one year but not in the past year), and current drinkers. Current drinkers were further subdivided into heavy (women: ≥3 drinks/day or ≥5 days/month with single-episode drinking of ≥4 drinks; men: ≥4 drinks/day or ≥5 days/month with single-episode drinking of ≥5 drinks), moderate (women: ≥2 drinks/day or ≥2 days/month with single-episode binge drinking; men: ≥3 drinks/day or ≥2 days/month with single-episode binge drinking), and light drinkers (women: ≤1 drink/day; men: ≤2 drinks/day) (25).

### 2.6 Statistical analysis

All analyses followed the guidelines of the NHANES dataset. The main sampling units, pseudo variances, and masked variances in sampling weights were adopted in multi-stage sampling designs, and nationally representative estimates were ensured. Given the intricate sampling structure of NHANES, a two-day dietary sample was chosen following appropriate weighting procedures (1/6 * WTDR2D). Median values were utilized to describe continuous variables (P25, P75), whereas categorical variables were manifested as numbers (percentages). In this study, pairwise comparisons of categorical variables were made through Pearson’s chi-square test. Since the continuous variables deviated from a normal distribution as the Kolmogorov–Smirnov test examined, non-parametric tests were utilized to assess group differences.

DI-GM was converted into categorical variables based on quartiles for statistical analysis, with the lowest quartile (Q1) as the control group. Initially, univariate and multivariate weighted logistic regression (LR) models, along with restricted cubic splines (RCS), were leveraged to gauge odds ratios (ORs) and 95% confidence intervals (CIs) to appraise the links between DI-GM and MetS and its components. Specifically, Model 1 was a crude (unadjusted) model. Model 2 was adjusted for gender, age, and race. Model 3 was further adjusted for education level, marital status, poverty income ratio (PIR), smoking status, medication history, total energy intake, and alcohol consumption. Subsequently, subgroup analyses were implemented to inspect the effects of key demographic and clinical variables on the link. All covariates, except those used for stratification, were adjusted in the models. These analyses were stratified by sex, age, race, education level, smoking status, and alcohol intake. The interactions between DI-GM and these variables were evaluated in LR models. Specifically, multiplicative interaction terms (DI-GM × covariate) were included to assess whether the association between dietary scores and MetS varied across subgroups. The significance of interactions was tested using analysis of variance. Mediation analysis was implemented to ascertain the mediating role of DII in the links of DI-GM with MetS and its components. Bootstrap methods with 1,000 simulations were used to estimate total, direct, and indirect effects, as well as the proportion mediated. Propensity score matching (PSM) was employed to diminish potential selection bias and confounding. Propensity scores were estimated using LR models incorporating relevant covariates. For the purpose of matching, participants in the highest DI-GM quartile (Q4) were designated as the “exposed group,” while those in the lower DI-GM quartiles (Q1, Q2, and Q3) formed the “unexposed group.” The covariates included in the propensity score model were the same as those adjusted in the multivariable logistic regression models. To prevent bias, the outcome variable, MetS, was excluded from the propensity score calculation. Participants were matched in a 1:2 ratio using the nearest neighbor matching algorithm based on these propensity scores. The balance of covariates after matching was appraised by comparing standardized mean differences (SMDs) between matched groups, with SMD < 0.1 indicating an acceptable balance. Weighted multivariate LR models were then utilized for the matched data, reducing potential bias and ensuring finding robustness. To further ensure the robustness of the associations between DI-GM and MetS, we conducted an additional analysis where (*N* = 16,225), building upon the covariates adjusted in Model 3, we further accounted for protein, total fat, and carbohydrate intake, as well as physical activity level. All statistical analyses were made in R 4.4.1 and MSTATA software (^1^). *p* < 0.05 inferred statistical significance.

## 3 Results

### 3.1 Baseline characteristics of the population

The baseline features are presented in Table 1. Among the 20,999 participants, the median age was 47 years, 51% were female, and the majority were White. MetS was present in 23% of the participants. After DI-GM was categorized into quartiles, Q1 was the lowest DI-GM group (0 ≤ DI-GM ≤ 4), Q2 was the relatively low DI-GM group (4 < DI-GM ≤ 5), Q3 was the relatively high DI-GM group (5 < DI-GM ≤ 6), and Q4 was the highest DI-GM group (6 < DI-GM ≤ 12). MetS prevalence in Q1, Q2, Q3, and Q4 was 25, 24, 21, and 21%, respectively. Notable differences were revealed across the four DI-GM groups in sex, age, race, PIR, education, marital status, smoking, alcohol intake, medication history, FPG, HDL-C, TG, WC, BMI, and DII (*p* < 0.05).

**Table 1:** Baseline characteristics of the participants.

Characteristic	Overall^b^	Q1^b^	Q2^b^	Q3^b^	Q4^b^	*p*-value^c^
Gender (%)						<0.001
Female	10,642(51%)	3,740(47%)	2,427(51%)	2061(52%)	2,414(56%)	
Male	10,357(49%)	4,187(53%)	2,353(49%)	1881(48%)	1936(44%)	
Age, years	47(33,60)	44(31,58)	47(33,59)	48(33,60)	51(37,62)	<0.001
Race (%)						<0.001
White	9,503(69%)	3,261(64%)	2098(68%)	1909(72%)	2,235(75%)	
Black	4,319(10%)	1993(14%)	1,005(10%)	713(8.9%)	608(5.9%)	
Mexican American	2,933(8.1%)	1,171(9.3%)	727(8.9%)	557(7.5%)	478(5.8%)	
Other	4,244(13%)	1,502(13%)	950(12%)	763(12%)	1,029(13%)	
Education (%)						<0.001
Less than 9th grade	1,672(4.0%)	745(5.3%)	372(3.7%)	307(3.9%)	248(2.5%)	
9-11th grade	2,732(9.4%)	1,255(12%)	660(9.9%)	459(8.1%)	358(6.0%)	
High school graduate/equivalent	4,782(23%)	2079(27%)	1,136(24%)	802(20%)	765(16%)	
College graduate or above	11,813(64%)	3,848(55%)	2,612(63%)	2,374(68%)	2,979(76%)	
PIR (%)						<0.001
<=1.0	4,185(14%)	1938(18%)	985(14%)	707(12%)	555(8.3%)	
1.0–3.0	8,730(35%)	3,555(38%)	1982(35%)	1,595(34%)	1,598(30%)	
>3.0	8,084(51%)	2,434(44%)	1813(51%)	1,640(54%)	2,197(62%)	
Smoking status (%)						<0.001
Former	5,235(25%)	1881(23%)	1,146(24%)	970(23%)	1,238(30%)	
Never	11,628(56%)	4,144(53%)	2,650(57%)	2,293(59%)	2,541(58%)	
Now	4,136(19%)	1902(23%)	984(19%)	679(17%)	571(13%)	
Alcohol consumption (%)						<0.001
Former	3,301(13%)	1,305(13%)	739(13%)	641(13%)	616(12%)	
Mild	7,464(38%)	2,558(33%)	1,626(36%)	1,449(39%)	1831(45%)	
Moderate	3,341(18%)	1,234(17%)	771(19%)	621(18%)	715(19%)	
Heavy	4,131(21%)	1807(26%)	985(22%)	718(20%)	621(15%)	
Never	2,762(10%)	1,023(11%)	659(11%)	513(10%)	567(9.3%)	
Medication history (%)	12,475(59%)	4,506(56%)	2,808(60%)	2,387(59%)	2,774(62%)	<0.001
MetS (%)	5,582(23%)	2,179(25%)	1,294(24%)	1,038(21%)	1,071(21%)	<0.001
Elevated FPG (%)	6,347(26%)	2,483(27%)	1,436(26%)	1,186(25%)	1,242(23%)	0.011
Low HDL-C (%)	6,377(29%)	2,549(31%)	1,484(30%)	1,178(29%)	1,166(25%)	<0.001
Elevated TG (%)	2,527(11%)	956(12%)	593(12%)	487(11%)	491(9.6%)	0.012
Elevated WC (%)	12,241(57%)	4,720(59%)	2,848(60%)	2,285(55%)	2,388(53%)	<0.001
Elevated BP (%)	8,270(34%)	3,162(35%)	1859(34%)	1,519(33%)	1730(34%)	0.7
BMI, kg/m2	28(24,33)	29(25,33)	28(25,33)	28(24,32)	27(24,32)	<0.001
Kcal	2108.89(2092.70,2125.09)	2087.94(2060.11,2115.77)	2112.04(2081.80,2142.28)	2108.92(2069.34,2148.49)	2138.09(2106.87,2169.30)	0.14
DII	1.56(−0.02,2.83)	2.34(1.00,3.31)	1.66(0.24,2.88)	1.12(−0.34,2.55)	0.35(−0.97,1.83)	<0.001

PIR, Poverty-income ratio; MetS, Metabolic Syndrome; FPG, Fasting Plasma Glucose; HDL-C, High-Density Lipoprotein Cholesterol; TG, Triglycerides; WC, Waist Circumference; BP, Blood Pressure; BMI, Body Mass Index; DII, Dietary Inflammatory Index.

b n (unweighted) (%); Median(Q1, Q3).

c Pearson’s X^2: Rao and Scott adjustment; Design-based Kruskal-Wallis test.

### 3.2 Link between DI-GM and MetS

Significant associations were revealed in Model 1 (unadjusted crude model) (OR = 0.94, 95% CI: 0.91–0.97) and Model 2 (OR = 0.90, 95% CI: 0.88–0.93). In Model 3, a significant negative correlation between DI-GM and MetS persisted (OR = 0.92, 95% CI: 0.89–0.95) (all *p* < 0.001). For every one-unit increase in DI-GM, MetS prevalence decreased by 8%. Furthermore, when DI-GM was a categorical variable (quartiles), the Q4 exhibited a 20% reduced odds of MetS compared to Q1 (OR = 0.80, 95% CI: 0.69–0.92), with a P for trend = 0.02 (Table 2).

**Table 2:** The correlation between DI-GM and the prevalence of metabolic syndrome in the study participants.

Variable	Model 1		Model 2		Model 3	
	OR(95%CI)	*P*	OR(95%CI)	*P*	OR(95%CI)	*P*
MetS						
DI-GM Continuous	0.94(0.91,0.97)	<0.001	0.90(0.88,0.93)	<0.001	0.92(0.89,0.95)	<0.001
Quartile 1	Ref.		Ref.		Ref.	
Quartile 2	0.95(0.85,1.07)	0.4	0.90(0.80,1.02)	0.093	0.93(0.80,1.09)	0.4
Quartile 3	0.82(0.72,0.93)	0.002	0.73(0.63,0.83)	<0.001	0.83(0.71,0.97)	0.021
Quartile 4	0.78(0.69,0.89)	<0.001	0.64(0.56,0.73)	<0.001	0.80(0.69,0.92)	0.003
P for trend	0.002		<0.0001		0.021	
Elevated FPG						
DI-GM Continuous	0.95(0.92,0.98)	<0.001	0.93(0.90,0.95)	<0.001	0.94(0.91,0.96)	<0.001
Quartile 1	Ref.		Ref.		Ref.	
Quartile 2	0.92(0.81,1.05)	0.2	0.91(0.80,1.05)	0.2	0.90(0.79,1.03)	0.11
Quartile 3	0.88(0.78,1.00)	0.047	0.85(0.75,0.97)	0.019	0.85(0.75,0.98)	0.023
Quartile 4	0.81(0.71,0.91)	0.001	0.74(0.65,0.84)	<0.001	0.73(0.64,0.83)	<0.001
P for trend	0.097		0.027		0.049	
Low HDL-C						
DI-GM Continuous	0.93(0.91,0.96)	<0.001	0.92(0.90,0.95)	<0.001	0.95(0.92,0.98)	<0.001
Quartile 1	Ref.		Ref.		Ref.	
Quartile 2	0.93(0.82,1.06)	0.3	0.92(0.80,1.05)	0.2	0.95(0.83,1.09)	0.5
Quartile 3	0.88(0.77,1.01)	0.06	0.87(0.76,0.99)	0.035	0.92(0.81,1.05)	0.2
Quartile 4	0.71(0.62,0.82)	<0.001	0.69(0.61,0.79)	<0.001	0.78(0.68,0.88)	<0.001
P for trend	0.023		0.019		0.125	
Elevated WC						
DI-GM Continuous	0.94(0.92,0.96)	<0.001	0.89(0.87,0.92)	<0.001	0.90(0.88,0.93)	<0.001
Quartile 1	Ref.		Ref.		Ref.	
Quartile 2	1.03(0.92,1.15)	0.6	0.97(0.86,1.08)	0.5	0.96(0.85,1.08)	0.5
Quartile 3	0.85(0.77,0.95)	0.003	0.77(0.69,0.85)	<0.001	0.77(0.69,0.86)	<0.001
Quartile 4	0.78(0.70,0.87)	<0.001	0.63(0.56,0.72)	<0.001	0.63(0.56,0.72)	<0.001
P for trend	<0.0001		<0.0001		<0.0001	
Elevated BP						
DI-GM Continuous	0.99(0.96,1.01)	0.4	0.93(0.90,0.96)	<0.001	0.94(0.91,0.96)	<0.001
Quartile 1	Ref.		Ref.		Ref.	
Quartile 2	0.99(0.89,1.10)	0.9	0.93(0.83,1.04)	0.2	0.88(0.78,1.00)	0.046
Quartile 3	0.94(0.82,1.07)	0.3	0.80(0.68,0.95)	0.012	0.79(0.66,0.95)	0.015
Quartile 4	0.99(0.88,1.12)	0.9	0.75(0.65,0.86)	<0.001	0.75(0.64,0.86)	<0.001
P for trend	0.444		0.005		0.035	
Elevated TG						
Quartile 1	1.10(0.94,1.29)	0.2	1.16(0.98,1.36)	0.076	1.10(0.93,1.31)	0.3
Quartile 2	1.13(0.95,1.35)	0.2	1.15(0.96,1.38)	0.12	1.13(0.93,1.35)	0.2
Quartile 3	Ref.		Ref.		Ref.	
Quartile 4	0.85(0.70,1.03)	0.1	0.82(0.68,0.99)	0.042	0.84(0.69,1.01)	0.061

Data are presented as weighted odds ratios (OR) with 95% confidence intervals (CI). Model 1 is the crude model. Model 2 is adjusted for gender, age, race. Model 3 is further adjusted for education, marital relations, PIR, smoking, medication history, energy and alcohol consumption.

Additionally, the links between DI-GM and MetS-related biochemical indicators are presented in Table 2. Using multivariate LR analysis, DI-GM was significantly negatively associated with elevated FPG, WC, BP, and reduced HDL-C levels. However, no significant association was observed with TG. Notably, WC showed a notable negative link with DI-GM in the Q3 and Q4 groups across all three models (*p* < 0.001, P for trend < 0.0001), suggesting that a healthy dietary pattern is conducive to gut microbiota diversity and may effectively improve abdominal obesity, thereby reducing the overall odds of MetS. For BP, the likelihood of elevated BP was reduced by 12% (OR = 0.88, 95% CI: 0.78–1.00), 21% (OR = 0.79, 95% CI: 0.66–0.95), and 25% (OR = 0.75, 95% CI: 0.64–0.86) in the Q2, Q3, and Q4, respectively. A similar trend of reduced odds was observed for FPG, indicating that optimizing dietary structure and improving gut microbiota diversity may diminish the odds of hypertension and diabetes, highlighting dietary intervention as an effective strategy for managing MetS. Interestingly, in Model 2 for TG, the Q4 group showed an 18% reduced odds of MetS in contrast to the Q3 group (OR = 0.82, 95% CI: 0.68–0.99), but this link was not pronounced in Model 3.

### 3.3 RCS analysis

**Figure 2:**
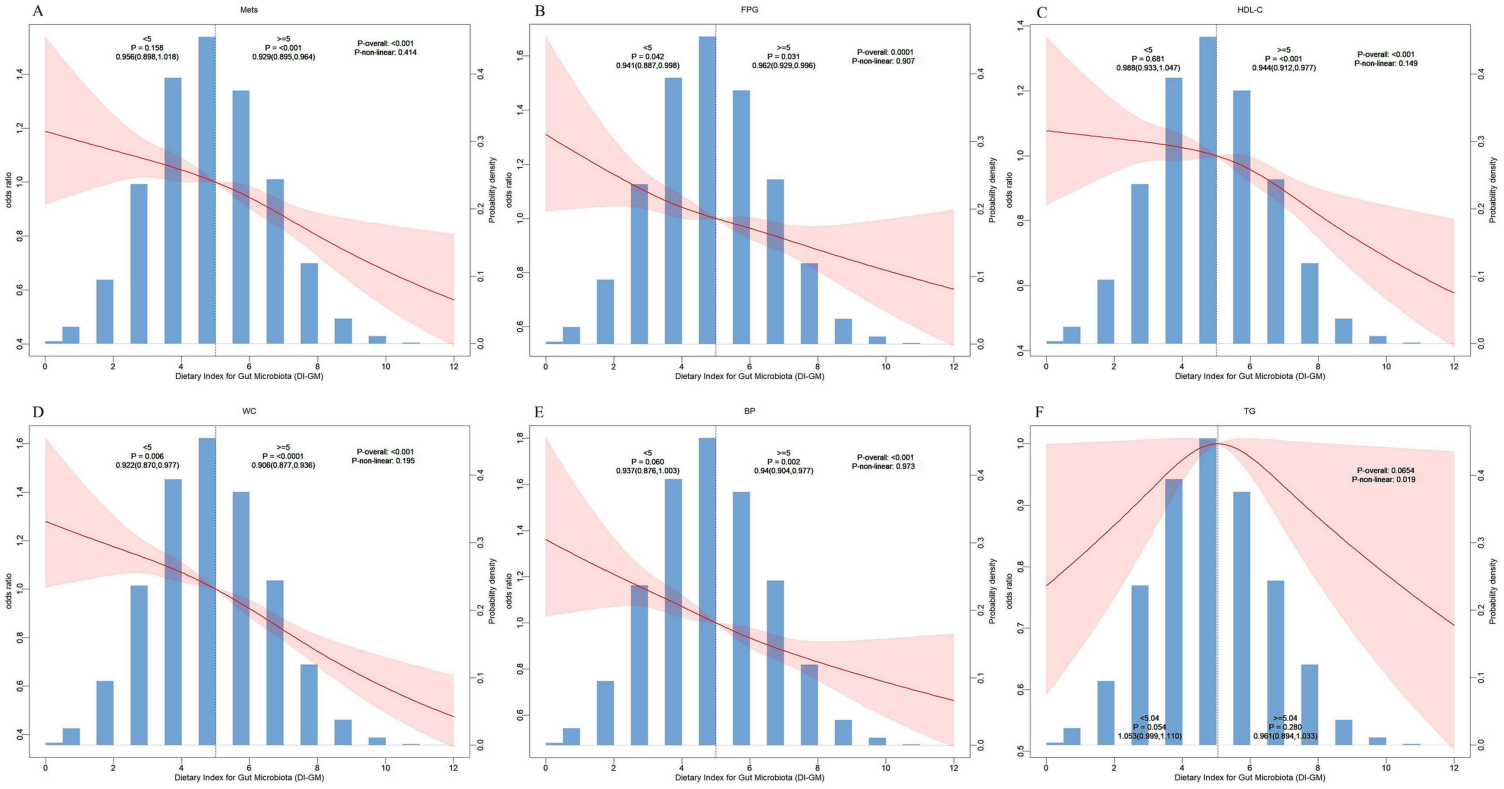
The restricted cubic spline curves for the association between DI-GM and metabolic syndrome and its components. **(A)**, metabolic syndrome; **(B)**, hyperglycemia; **(C)**, low HDL-C; **(D)**, central obesity; **(E)**, hypertension; **(F)**, hypertriglyceridemia.

A nonlinear link between DI-GM and MetS was investigated through multivariate-adjusted RCS analysis. As illustrated in Figure 2A, a notable linear negative link was revealed between DI-GM and MetS (P for nonlinear = 0.41). Further analysis of individual MetS components revealed consistent linear negative associations between DI-GM and FPG (Figure 2B), HDL-C (Figure 2C), WC (Figure 2D), and BP (Figure 2E) (P for nonlinear = 0.91, 0.15, 0.20, and 0.97, respectively). Notably, the dose–response curve for DI-GM and TG demonstrated an inverted U-shaped pattern (Figure 2F). The odds of elevated TG increased with elevated DI-GM levels; however, when DI-GM exceeded a threshold of 5.04, the OR for elevated TG exhibited a declining trend with further increases in DI-GM. This trend, however, did not achieve statistical significance (P-overall: 0.07).

### 3.4 Mediation analysis

In this model, DI-GM was the independent variable, MetS the outcome variable, and DII the mediator. A significant indirect association of DI-GM on MetS through DII was observed, with an estimate of-0.00265 (95% CI: −0.00445, −0.00167). This finding suggested that DII partially mediated the link between DI-GM and MetS. Furthermore, after adjusting for DII, DI-GM continued to exert a significant direct inhibitory association with MetS, with a direct association estimate of-0.00763 (95% CI: −0.01181, −0.00425). This indicated that DI-GM influenced MetS through both direct and indirect pathways. Approximately 25.8% of the total association was mediated by DII (Table 3).

**Table 3:** Mediation analysis of the association between dietary index for gut microbiota and metabolic syndrome components, mediated by dietary inflammatory index.

Metabolic syndrome component	Independent variable	Mediator	Total effect		Indirect effect		Direct effect		Proportion mediated, % (95% CI)
			Coefficient (95% CI)	*p* value	Coefficient (95% CI)	*p* value	Coefficient (95% CI)	*P* value	
MetS	DI-GM	DII	−0.01028 (−0.01430, −0.00727)	<0.001	−0.00265 (−0.00445, −0.00167)	<0.001	−0.00763 (−0.01181, −0.00425)	<0.001	25.8 (14.4, 46.2)
Elevated FPG	DI-GM	DII	−0.00897 (−0.01261, −0.00576)	<0.001	−0.00198 (−0.00342, −0.00049)	<0.001	−0.00698 (−0.01078, −0.00276)	<0.001	22.1 (5.7, 51.5)
Low HDL-C	DI-GM	DII	−0.00876 (−0.01292, −0.00540)	<0.001	−0.00472 (−0.00640, −0.00366)	<0.001	−0.00404 (−0.00853, −0.00042)	0.02	53.9 (33.6, 92.3)
Elevated WC	DI-GM	DII	−0.01528 (−0.01887, −0.01163)	<0.001	−0.00525 (−0.00649, −0.00378)	<0.001	−0.01003 (−0.01386, −0.00635)	<0.001	34.4 (22.3, 46.9)
Elevated BP	DI-GM	DII	−0.00962 (−0.01267, −0.00685)	<0.001	−0.00069 (−0.00187, 0.00032)	0.18	−0.00893 (−0.01190, −0.00589)	<0.001	7.2 (−3.3, 19.2)
Elevated TG	DI-GM	DII	−0.00026 (−0.00282, 0.00212)	0.8	−0.00059 (−0.00130, 0.00038)	0.22	0.00033 (−0.00285, 0.00276)	0.78	N/A^a^

a The mediated proportion for elevated triglycerides (TG) is not reported. Although a numerical proportion could be mathematically calculated, given that the total association between DI-GM and TG was not statistically significant and very close to zero, the resulting percentage would be highly unstable and uninterpretable (e.g., yielding disproportionately large or negative values).

Additionally, Table 3 presents the mediating associations of DII between DI-GM and various components of MetS. Specifically, DII demonstrated similar direct and indirect mediating associations between DI-GM and FPG, HDL-C, and WC, with respective mediation proportions of 22.1, 53.9, and 34.4%. Notably, although DI-GM exhibited a significant inhibitory association with BP, with a direct association estimate of-0.00893 (95% CI: −0.01190, −0.00589), no marked indirect association of DII was noted between the two. Similarly, no direct or indirect mediating association of DII was found between DI-GM and TG. Although a numerical proportion mediated could be mathematically calculated, given that the total association between DI-GM and TG was not statistically significant and very close to zero, the resulting percentage would be highly unstable and uninterpretable. Therefore, this proportion is not reported to prevent misunderstanding.

### 3.5 Subgroup analyses

**Figure 3:**
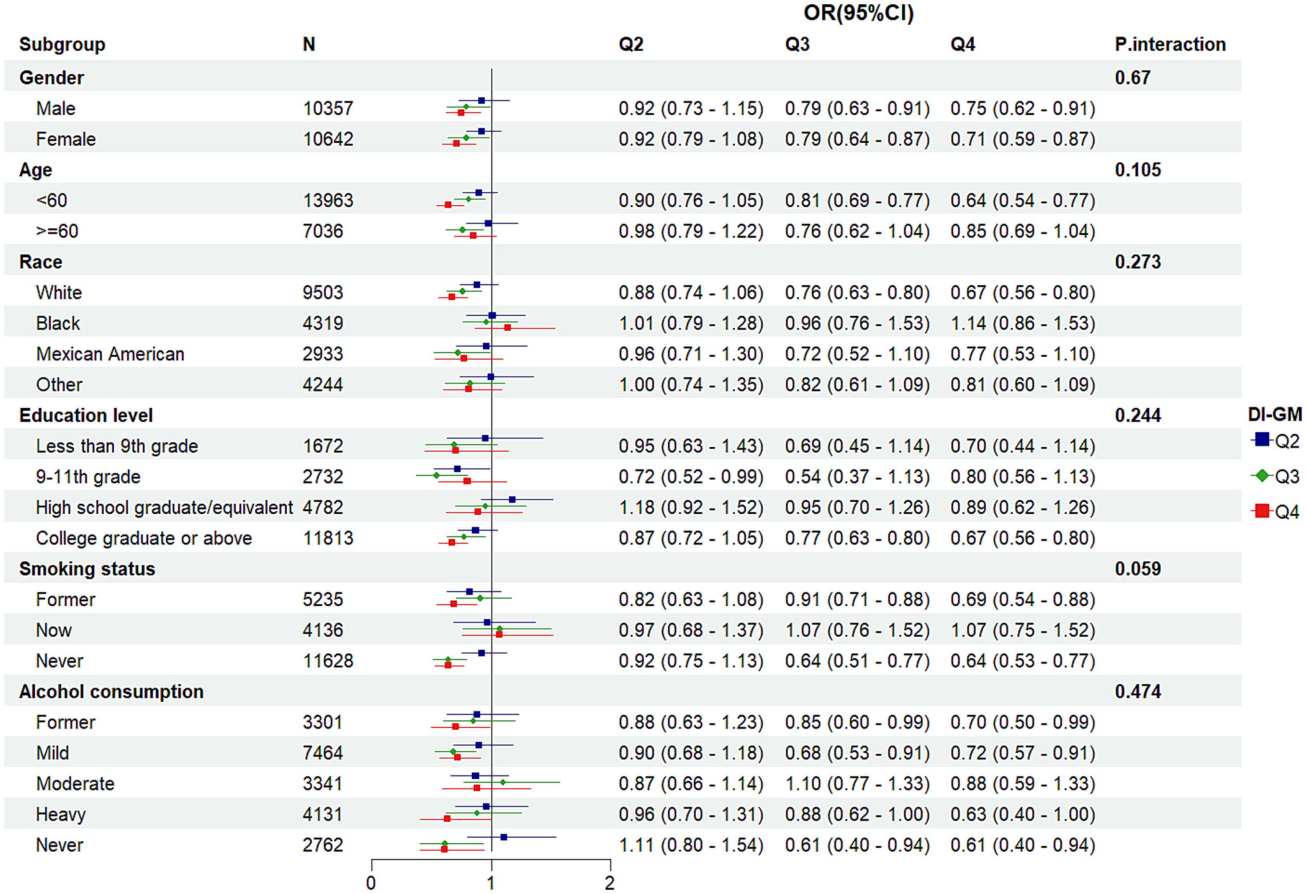
Subgroup analysis of the association between DI-GM and metabolic syndrome.

To elucidate the consistency of the association across distinct demographic and behavioral subgroups, a detailed subgroup analysis was conducted. A LR model was then applied to these subgroups, with all covariates, excluding those used for stratification, being adjusted. Higher levels of DI-GM (Q4) were greatly inversely linked with MetS prevalence in various subgroups, including males (OR = 0.75, 95% CI: 0.62–0.91), females (OR = 0.71, 95% CI: 0.59–0.87), individuals aged less than 60 years (OR = 0.64, 95% CI: 0.54–0.77), White individuals (OR = 0.67, 95% CI: 0.56–0.80), college graduates (OR = 0.67, 95% CI: 0.56–0.80), former smokers (OR = 0.69, 95% CI: 0.54–0.88), never smokers (OR = 0.64, 95% CI: 0.53–0.77), former drinkers (OR = 0.70, 95% CI: 0.50–0.99), light drinkers (OR = 0.72, 95% CI: 0.57–0.91), and never drinkers (OR = 0.61, 95% CI: 0.40–0.94). Importantly, interaction *p*-values revealed no statistically significant interactions between DI-GM and the stratified variables across the subgroups (P interaction >0.05) (Figure 3), indicating that the inverse association between DI-GM and MetS did not significantly differ across these groups.

### 3.6 Sensitivity analysis

To control for the influence of confounding factors, PSM analysis was employed, followed by univariate and multivariate LR models. These models were utilized to adjust for various potential confounders in the original (unmatched) data, yielding results consistent with the primary estimates reported. The post-matching analysis elicited a notable negative link between DI-GM and MetS in Model 3 (OR = 0.92, 95% CI: 0.90–0.95, *p* < 0.001). The Q4 manifested a 31% reduced odds of developing MetS compared to Q1 [OR = 0.69 (95% CI: 0.60, 0.80)], with a P for trend <0.001. Additionally, the links between DI-GM and five MetS-related biochemical indicators across different models post-matching demonstrated similar outcomes, further substantiating the finding robustness (Supplementary Table 3). Additionally, to further enhance the robustness of our findings, we conducted another sensitivity analysis where we additionally adjusted for macronutrient intake (protein, total fat, and carbohydrates) and physical activity in Model 3. The results from this analysis remained consistent with our primary findings (Supplementary Table 4).

## 4 Discussion

This paper first systematically assessed the link between DI-GM and the odds of MetS while also investigating the mediating role of DII. The results demonstrated that a high DI-GM score was greatly correlated with a reduced odds of MetS. This correlation persisted after adjusting for confounders, with consistent findings in both continuous and categorical DI-GM analyses. DII partially mediated the link, underscoring the intricate connections among gut microbiota, dietary inflammation, and MetS.

The link between gut microbiota and MetS has attracted considerable attention in recent years. Studies have regarded gut microbiota dysbiosis as a crucial factor in MetS pathogenesis, particularly when influenced by dietary and inflammatory factors that alter the composition and functionality of gut microbiota. For instance, Kruttika Dabke et al. (6) have established a strong association between gut microbiota and MetS. Specifically, high-fat diets can compromise the intestinal barrier, allowing bacterial products such as lipopolysaccharides to enter the bloodstream, thereby inducing low-grade chronic inflammation (LGCI). This metabolic endotoxemia significantly impairs insulin sensitivity. Moreover, Perler et al. (26) have examined the intricate interactions among diet, gut microbiota, and host health, noting that Western diets—with high fat and low fiber—often result in gut microbiota dysbiosis, thereby elevating the odds of obesity, MetS, and cardiovascular diseases. Studies in both human and animal models have revealed that gut microbiota profoundly influences host metabolism through metabolites like short-chain fatty acids (SCFAs). Boulangé et al. (27). have emphasized that gut microbiota is crucial in host energy metabolism and obesity and metabolic diseases through interactions with the host immune system. Alterations in gut microbiota can affect host metabolic health by promoting LGCI, fat accumulation, and insulin resistance. Additionally, Croci et al. (28) have demonstrated that certain gut microbial metabolites, including lipopolysaccharides, indoxyl sulfate, and p-cresyl sulfate, can initiate LGCI, a fundamental pathological mechanism underlying MetS.

DI-GM is a novel tool for the systematic evaluation of the link between gut microbiota and MetS. However, the precise connection between DI-GM and MetS is unclear. Our study demonstrated that an increased DI-GM score was greatly linked with a reduced odds of MetS. Similarly, Zouiouich et al. (29), in an analysis of the Northern Finland Birth Cohort 1966 and the TwinsUK cohort, observed that higher gut microbiota *α*-diversity was closely associated with lower insulin resistance, better glycemic control, and reduced C-reactive protein levels. The impact of gut microbiota diversity on metabolic health is likely mediated through energy metabolism, gut barrier function, and systemic inflammation. Notably, in the pathogenesis of insulin resistance, gut microbiota alleviates LGCI by modulating SCFA production and balancing immune responses. Furthermore, Gantenbein and Kanaka-Gantenbein (30) explored the beneficial effects of the Mediterranean diet (rich in fruits, nuts, vegetables, whole grains, and olive oil) on gut microbiota and metabolic health. The Mediterranean diet enhanced gut microbiota diversity via its antioxidant and anti-inflammatory properties. Its high fiber and polyphenol content regulate microbiota, promote SCFA production, improve insulin sensitivity, and lower odds of MetS. Together, these studies and our findings strongly support the pivotal role of gut microbiota in mitigating the odds of MetS, further corroborating the influence of microbiota diversity and dietary patterns on MetS management.

The significant inverse association observed between the DI-GM score and the odds of MetS is underpinned by complex and multifaceted biological mechanisms. Unlike general dietary indices that provide an overall health score, DI-GM’s strength lies in its ability to pinpoint how specific dietary components influence gut microbiota. These mechanisms primarily involve the precise modulation of gut microbial composition, function, and their metabolic byproducts by specific dietary components within the DI-GM, which, in turn, directly influences host metabolic health.

Beneficial components of the DI-GM, such as dietary fiber, whole grains, and specific plant-based foods like broccoli and soy, are recognized as crucial modulators of the gut microbiota. For instance, dietary fiber and whole grains serve as primary substrates for gut microbial fermentation. This process significantly promotes the production of acetate, propionate, and butyrate by enriching short-chain fatty acid (SCFA)-producing bacteria (e.g., *Roseburia, Faecalibacterium*) and providing specific carbohydrates (e.g., *β-glucans, arabinoxylans*) (31). Butyrate, an important energy source for colonocytes, has been demonstrated to exert significant anti-inflammatory effects through the inhibition of the NF-κB signaling pathway, thereby reducing systemic inflammation and enhancing insulin sensitivity (32). Similarly, glucosinolates, abundant in cruciferous vegetables like broccoli, are metabolized by gut microbes into isothiocyanates. These compounds are known for their anti-inflammatory and antioxidant properties and can influence inflammatory pathways by modulating gut microbiota structure or directly acting on host cells (33–35). Soy, rich in isoflavones and dietary fiber, contributes to the growth of beneficial bacteria such as Bifidobacterium and Lactobacillus, leading to the production of metabolites with anti-inflammatory and antioxidant properties. Equol, a metabolite of soy isoflavones, may also regulate gut microbiota and affect lipid metabolism and inflammatory responses (36). Furthermore, fermented dairy products directly supplement probiotics, aiding in the maintenance of gut microbial balance and producing lactic acid and acetic acid. These acids contribute to maintaining intestinal pH, inhibiting the growth of harmful bacteria, and exerting anti-inflammatory effects through the regulation of cytokine production (37–39).

Conversely, detrimental components within the DI-GM, including processed meats, red meat, refined grains, and high-fat diets, are hypothesized to promote metabolic dysfunction through distinct microbial pathways. For instance, high consumption of red and processed meats (which are typically rich in saturated fats and heme iron) has been strongly associated with altered gut microbial metabolism of dietary L-carnitine and phosphatidylcholine, leading to the production of trimethylamine N-oxide (TMAO) precursors (40, 41). TMAO, an atherogenic metabolite, is linked to an increased odds of cardiovascular disease and has been shown to promote inflammation and plaque formation (42). Refined grains, which lack fermentable fibers, and high-fat diets often result in a reduction of beneficial gut bacteria and an increase in pro-inflammatory species (43, 44). This dietary pattern is considered to severely compromise the integrity of the intestinal barrier, thereby exacerbating metabolic endotoxemia. Metabolic endotoxemia, as demonstrated by studies such as that by Chmielarz et al. (45), results from increased translocation of bacterial lipopolysaccharide (LPS) from the intestinal lumen into systemic circulation. LPS, a potent pro-inflammatory molecule from the cell wall of Gram-negative bacteria, activates host immune responses upon entering the circulatory system, thereby driving chronic low-grade inflammation (LGCI). This LGCI is a proposed underlying pathological mechanism for MetS, impairing insulin sensitivity and promoting adiposity and the progression of MetS. These findings, together with our own, collectively support the DI-GM as a comprehensive indicator effectively reflecting the impact of diet on gut microbiota health.

The DI-GM score is designed to indirectly reflect the impact of dietary patterns on the gut microbiome by evaluating specific dietary components known to exert either positive or negative influences on gut microbial composition, diversity, and function (46). This index integrates the intake of various beneficial components to generate a quantitative score. Consequently, the DI-GM serves as a valuable, mechanistic proxy indicator of dietary quality on gut microbiota health. Its focus is on the shaping effect of diet on the overall microbial environment, providing a unique dietary lens to investigate the microbiota-host axis, rather than on the direct measurement of specific microbial species or their abundance (9). A higher DI-GM score indicates that an individual tends to consume more foods that support the growth of beneficial gut bacteria, promote microbial diversity, and maintain intestinal barrier integrity, which is closely associated with a healthy metabolic state (47). However, it is crucial to acknowledge that the actual composition and function of an individual’s gut microbiota are influenced by a multitude of factors beyond just dietary intake, including genetics, medication use (e.g., antibiotics), stress, physical activity, and environmental exposures. Therefore, while a higher DI-GM score indicates a diet conducive to gut health, it does not unequivocally guarantee an optimal microbial profile, nor does it fully capture the complex interplay of all factors shaping the gut microbiota. The impact of diet on gut microbiota is also dynamic and depends significantly on the duration and consistency of adherence to a particular dietary pattern, as well as the interplay with an individual’s unique metabolic and lifestyle factors.

The DII, which measures the inflammatory potential of a diet, has been widely utilized to assess the link between diet and the odds of chronic disease (48). DII was chosen as a key mediator variable, primarily due to its pivotal role in inflammation regulation. The onset and progression of MetS are closely linked to systemic LGCI. Gut microbiota directly influences host inflammation by metabolizing its substrates into SCFAs, lipopolysaccharides, and other metabolic products (49–51). DI-GM reflects the dietary modulation of the gut microbiome, indirectly influencing the inflammatory state. As a tool that integrates the inflammatory effects of dietary components, DII captures the overall impact of DI-GM-driven dietary patterns on inflammatory pathways, thereby regulating inflammation-mediated mechanisms associated with MetS. Consequently, we proved that DII mediated the link between DI-GM and MetS. Existing research supports that an elevated DII increases the odds of MetS. For instance, Szypowska et al. (52) demonstrated that higher DII correlated with MetS prevalence, particularly with components such as serum TG, abdominal obesity, and hypertension. Pro-inflammatory dietary components, such as foods rich in sugar and fat, may contribute to MetS by increasing LGCI. Similarly, Kenđel Jovanović et al. (53) stated that people with higher DII scores were more prone to developing key MetS components such as abdominal obesity, hypertension, and elevated TG. Furthermore, Canto-Osorio et al. (54) highlighted that a higher DII was significantly associated with the odds of MetS in Mexican adults. Notably, as DII scores increased, the likelihood of participants developing insulin resistance and elevated blood glucose also increased.

The uniqueness of the DI-GM lies in its direct focus on the impact of diet on gut microbiota, which provides a novel perspective for the development of personalized nutritional strategies and public health guidelines. Specifically, based on the components of DI-GM, simplified dietary assessment tools or mobile applications can be developed to allow the public to conveniently assess the potential benefits or risks of their diet on gut microbiota. For example, by encouraging increased intake of beneficial components such as avocados and whole grains, and limiting harmful components such as processed meats and refined grains, individuals can be provided with specific, actionable dietary improvement recommendations. Compared with existing dietary indices (e.g., DII or HEI), the DI-GM may offer greater ease of use due to its focus on a relatively small number of dietary components (14 types). This suggests greater potential for its promotion in clinical practice and public health education. Simultaneously, the DI-GM provides a new dimension for evaluating dietary patterns from the perspective of gut microbiota-host interactions, which differs from traditional assessments based on macronutrients or single food groups. Future research should further explore the independent predictive ability of DI-GM in forecasting MetS and other chronic diseases. Additionally, prospective studies are warranted to validate its effectiveness in guiding long-term dietary interventions, thereby providing more robust evidence for its translation into a widely applied public health tool.

This paper offers certain advantages. First, it relies on the nationally representative NHANES sample, with large sample size and multiple cycles, ensuring high statistical power and broad applicability. Second, DI-GM, as an innovative dietary index, is examined for the first time concerning MetS in our study. Additionally, we explored the link between DI-GM and various MetS subtypes, providing separate interpretations of these results. This approach highlights the rigor of our research. Furthermore, the DI-GM offers a more targeted approach for personalized nutritional recommendations aiming to optimize gut microbiota and metabolic health. Third, many confounders were adjusted to minimize bias, and result robustness was validated through sensitivity analysis. However, there are certain shortcomings. First, cross-sectional design restrains us illustrating causality. Longitudinal studies are warranted to clarify the causality between DI-GM and MetS. Second, dietary intake was recorded through 24-h recalls, which may be subject to recall bias. Moreover, the DI-GM, while developed based on absolute intake, was not subjected to energy adjustment. While DI-GM can assess diet quality related to gut microbiota, it may not totally reflect the complex influence of all dietary factors on gut microbiota. Lastly, despite adjustment for various covariates, residual confounders may remain.

## 5 Conclusion

This paper reveals a notable negative link between DI-GM and MetS. DII mediates the link between DI-GM and MetS. This finding offers new evidence for understanding how diet, through its regulation of gut microbiota and inflammation, collectively influences metabolic health. It suggests that improving dietary quality to enhance gut microbiota and reduce inflammation may be effective for MetS prevention. Furthermore, these results provide scientific support for public health initiatives focused on promoting healthy dietary interventions.

## Data Availability

The datasets presented in this study can be found in online repositories. The names of the repository/repositories and accession number(s) can be found below: https://wwwn.cdc.gov/nchs/nhanes/default.aspx.

## Footnotes

^1^ www.mstata.com
