# Supplementary material for: Association between the dietary index for gut microbiota and metabolic syndrome: the mediating role of the dietary inflammatory index
Source: Front Nutr. 2025 Jul 21;12:1617287. doi: 10.3389/fnut.2025.1617287 (PMC12318735; doi:10.3389/fnut.2025.1617287)
Supplement: Supplementary file 1 [file Table_1.docx]

Supplementary Table 1. DI-GM Components, Scoring Criteria, and FNDDS Classification Methods.

| **Componet** | **Included Foods within the Componet** | **Scoring** | **Method for Identifying and Quantifying from FNDDS** |
| --- | --- | --- | --- |
| **Beneficial to gut miceobiota** |  | For each component, a score of 1 if consumption at or above the sex-specificmedian,else0 | Intake amounts for beneficial components are summed from identified FNDDS food codes. |
| Avocados | Avocados |  | Identified by FNDDS food codes and descriptions explicitly listing "avocado" as a standalone item (e.g., "avocado, raw") or a quantifiable ingredient in composite dishes (e.g., "guacamole"). |
| Broccoli | Broccoli |  | Identified by FNDDS food codes and descriptions for "broccoli" in various forms (e.g., raw, cooked). |
| Chickpea | Chickpea |  | Identified by FNDDS food codes and descriptions for "chickpea" or "garbanzo beans" (e.g., canned chickpeas, chickpea flour). |
| Coffee | Coffee |  | Identified by FNDDS food codes and descriptions for "coffee" (e.g., brewed coffee, instant coffee). |
| Cranberries | Cranberries |  | Identified by FNDDS food codes and descriptions for "cranberries" (e.g., raw cranberries, cranberry sauce, cranberry juice). |
| Fermented dairy | Yogurt cheese, kefir, sour cream, buttermilk |  | Identified by FNDDS food codes and descriptions for fermented dairy products (e.g., various types of yogurt, cheese, kefir, sour cream, buttermilk). |
| Fiber | Not applicable |  | Total dietary fiber intake calculated directly from FNDDS nutrient data (g/day). |
| Green tea | Green tea |  | Identified by FNDDS food codes and descriptions for "green tea." (Note: As mentioned in the original DI-GM paper, this component was often excluded from NHANES scoring if specific tea types were not recorded.) |
| Soybean | Soy products-Soy milk, Tofu |  | Identified by FNDDS food codes and descriptions for soy-based products (e.g., "soy milk," "tofu," "tempeh," "edamame"). |
| Whole grains | Grains defined as whole grains, containing the entire grain kernel-the bran, germ, and endosperm |  | Identified by FNDDS food codes and descriptions explicitly stating "whole grain" (e.g., "whole wheat bread," "oatmeal," "brown rice," "quinoa"). Classification adhered to USDA definitions for whole grains. |
| **Unfavorable to gut microbiota** |  | 0 if consumption at or above 40% energy from fat, else 1 | Intake amounts for unfavorable components are summed from identified FNDDS food codes. |
| Hight-fat diet(% energy) | Not applicable |  | Total fat intake calculated from FNDDS nutrient data (g/day), then converted to percentage of total energy intake. |
| Processed meat | Franjfurters, sausages, cornd beef, and luncheon meat that are made from beef, pork ,or poultry |  | Identified by FNDDS food codes and descriptions for "processed meats" (e.g., "frankfurters," "sausages," "corned beef," "luncheon meats"). Classification based on typical processing methods (curing, smoking, salting, or adding chemical preservatives). |
| Red meat | Beef, veal, pork, lamb, and game meat; excludes organ meat and cured meat |  | Identified by FNDDS food codes and descriptions for "red meats" (e.g., "beef," "pork," "lamb," "veal," "game meat"). Excludes organ meats and cured/processed red meats. |
| Refined grains | Refined grains that do not contain all of the components of the entire grain kernel |  | Identified by FNDDS food codes and descriptions for "refined grains" (e.g., "white bread," "white rice," "pasta made from refined flour"). Classification based on absence of bran and germ. |
