# Supplementary material for: Association between the dietary index for gut microbiota and metabolic syndrome: the mediating role of the dietary inflammatory index
Source: Front Nutr. 2025 Jul 21;12:1617287. doi: 10.3389/fnut.2025.1617287 (PMC12318735; doi:10.3389/fnut.2025.1617287)
Supplement: Supplementary file 2 [file Table_2.docx]

**Supplementary Table 2 Dietary composition parameters involved in DII,inflammatory effect scores, and intake values from the global composite data setc**

| Dietary composition parameter | Overall inflammatory effect score^b^ | Global daily mean intake(units/d) | Standard deviation of the global daily intake |
| --- | --- | --- | --- |
| Alcohol (g) | -0.278 | 13.98 | 3.72 |
| Vitamin B12 (μg) | 0.106 | 5.15 | 2.7 |
| Vitamin B6 (mg) | -0.365 | 1.47 | 0.74 |
| β-Carotene (μg) | -0.584 | 3718 | 1720 |
| Caffeine (g) | -0.11 | 8.05 | 6.67 |
| Carbohydrate (g) | 0.097 | 272.2 | 40 |
| Cholesterol (mg) | 0.11 | 279.4 | 51.2 |
| Energy (kcal) | 0.18 | 2056 | 338 |
| Total fat (g) | 0.298 | 71.4 | 19.4 |
| Fiber (g) | -0.663 | 18.8 | 4.9 |
| Folic acid (μg) | -0.19 | 273 | 70.7 |
| Iron (mg) | 0.032 | 13.35 | 3.71 |
| Magnesium (mg) | -0.484 | 310.1 | 139.4 |
| MUFA^d^(g) | -0.009 | 27 | 6.1 |
| Niacin (mg) | -0.246 | 25.9 | 11.77 |
| Protein (g) | 0.021 | 79.4 | 13.9 |
| PUFA^e^(g) | -0.337 | 13.88 | 3.76 |
| Vitamin B2 (mg) | -0.068 | 1.7 | 0.79 |
| Saturated fat (g) | 0.373 | 28.6 | 8 |
| Selenium (μg) | -0.191 | 67 | 25.1 |
| Vitamin B1 (mg) | -0.098 | 1.7 | 0.66 |
| Vitamin A (RE ^a^) | -0.401 | 983.9 | 518.6 |
| Vitamin C (mg) | -0.424 | 118.2 | 43.46 |
| Vitamin D (μg) | -0.446 | 6.26 | 2.21 |
| Vitamin E (mg) | -0.419 | 8.73 | 1.49 |
| Zinc (mg) | -0.313 | 9.84 | 2.19 |

^a^Retinol equivalents.

^b^Dietary composition parameter-specific overall inflammatory effect score.

^c^DII of a certain dietary component = (Daily intake of the dietary component - Global daily mean intake of the dietary component) / Standard deviation of the global daily intake for the dietary component * Overall inflammatory effect score of the dietary component. The DII for each participant was obtained by summing the DII of the 26 dietary components selected in this study.

^d^Monounsaturated fatty acids.

^e^Polyunsaturated fatty acids.
