# Supplementary material for: Association between the dietary index for gut microbiota and metabolic syndrome: the mediating role of the dietary inflammatory index
Source: Front Nutr. 2025 Jul 21;12:1617287. doi: 10.3389/fnut.2025.1617287 (PMC12318735; doi:10.3389/fnut.2025.1617287)
Supplement: Supplementary file 3 [file Table_3.docx]

**Supplementary Table 3.** Association Between DI-GM and Metabolic Syndrome Incidence After Propensity Score Matching Adjustment

| Variable | Model1 |  | Model2 |  | Model3 |  |
| --- | --- | --- | --- | --- | --- | --- |
|  | OR(95%CI) | P | OR(95%CI) | P | OR(95%CI) | P |
| MetS |  |  |  |  |  |  |
| DI-GM Continous | 0.92(0.89,0.95) | <0.001 | 0.91(0.88,0.94) | <0.001 | 0.92(0.90,0.95) | <0.001 |
| Quartile 1 | Ref. |  | Ref. |  | Ref. |  |
| Quartile 2 | 0.88(0.77,1.00) | 0.049 | 0.87(0.76,0.99) | 0.039 | 0.89(0.78,1.02) | 0.1 |
| Quartile 3 | 0.75(0.66,0.86) | <0.001 | 0.73(0.64,0.84) | <0.001 | 0.75(0.66,0.87) | <0.001 |
| Quartile 4 | 0.69(0.60,0.79) | <0.001 | 0.66(0.57,0.76) | <0.001 | 0.69(0.60,0.80) | <0.001 |
| P for trend | <0.001 |  | <0.0001 |  | <0.001 |  |
| Elevated FPG |  |  |  |  |  |  |
| DI-GM Continous | 0.93(0.91,0.96) | <0.001 | 0.92(0.89,0.95) | <0.001 | 0.93(0.90,0.96) | <0.001 |
| Quartile 1 | Ref. |  | Ref. |  | Ref. |  |
| Quartile 2 | 0.89(0.78,1.02) | 0.1 | 0.89(0.78,1.02) | 0.1 | 0.89(0.75,1.06) | 0.2 |
| Quartile 3 | 0.75(0.66,0.87) | <0.001 | 0.75(0.66,0.87) | <0.001 | 0.84(0.71,0.99) | 0.036 |
| Quartile 4 | 0.69(0.60,0.80) | <0.001 | 0.69(0.60,0.80) | <0.001 | 0.68(0.59,0.78) | <0.001 |
| P for trend | 0.038 |  | 0.012 |  | 0.028 |  |
| Low HDL-C |  |  |  |  |  |  |
| DI-GM Continous | 0.93(0.90,0.96) | <0.001 | 0.93(0.91,0.96) | <0.001 | 0.95(0.93,0.98) | 0.002 |
| Quartile 1 | Ref. |  | Ref. |  | Ref. |  |
| Quartile 2 | 0.86(0.75,1.00) | 0.048 | 0.86(0.75,1.00) | 0.048 | 0.91(0.77,1.07) | 0.2 |
| Quartile 3 | 0.74(0.62,0.88) | <0.001 | 0.74(0.62,0.88) | <0.001 | 0.93(0.80,1.08) | 0.3 |
| Quartile 4 | 0.73(0.63,0.85) | <0.001 | 0.73(0.63,0.85) | <0.001 | 0.78(0.68,0.90) | 0.001 |
| P for trend | 0.11 |  | 0.198 |  | 0.392 |  |
| Elevated WC |  |  |  |  |  |  |
| DI-GM Continous | 0.93(0.90,0.96) | <0.001 | 0.90(0.87,0.93) | <0.001 | 0.90(0.87,0.93) | <0.001 |
| Quartile 1 | Ref. |  | Ref. |  | Ref. |  |
| Quartile 2 | 0.97(0.85,1.11) | 0.6 | 0.97(0.85,1.11) | 0.6 | 0.93(0.81,1.06) | 0.3 |
| Quartile 3 | 0.79(0.69,0.91) | 0.002 | 0.79(0.69,0.91) | 0.002 | 0.74(0.64,0.86) | <0.001 |
| Quartile 4 | 0.71(0.62,0.82) | <0.001 | 0.71(0.62,0.82) | <0.001 | 0.63(0.54,0.73) | <0.001 |
| P for trend | <0.001 |  | <0.0001 |  | <0.0001 |  |
| Elevated BP |  |  |  |  |  |  |
| DI-GM Continous | 0.98(0.95,1.00) | 0.088 | 0.93(0.90,0.96) | <0.001 | 0.93(0.90,0.96) | <0.001 |
| Quartile 1 | Ref. |  | Ref. |  | Ref. |  |
| Quartile 2 | 0.93(0.81,1.06) | 0.3 | 0.93(0.81,1.06) | 0.3 | 0.86(0.75,1.00) | 0.048 |
| Quartile 3 | 0.74(0.64,0.86) | <0.001 | 0.74(0.64,0.86) | <0.001 | 0.74(0.62,0.88) | <0.001 |
| Quartile 4 | 0.63(0.54,0.73) | <0.001 | 0.63(0.54,0.73) | <0.001 | 0.73(0.63,0.85) | <0.001 |
| P for trend | 0.254 |  | 0.008 |  | 0.02 |  |
| Elevated TG |  |  |  |  |  |  |
| Quartile 1 | 1.22(1.03,1.45) | 0.024 | 1.23(1.03,1.46) | 0.024 | 1.20(0.99,1.44) | 0.059 |
| Quartile 2 | 1.20(0.99,1.45) | 0.068 | 1.19(0.98,1.45) | 0.077 | 1.20(0.98,1.46) | 0.073 |
| Quartile 3 | Ref. |  | Ref. |  | Ref. |  |
| Quartile 4 | 0.90(0.75,1.09) | 0.3 | 0.90(0.75,1.09) | 0.3 | 0.92(0.76,1.11) | 0.4 |

**Notes:** Data are presented as weighted odds ratios (OR) with 95% confidence intervals (CI). Model 1 is the crude model. Model 2 is adjusted for gender, age, race. Model 3 is further adjusted for education, marital relations, PIR, smoking, medication history, energy and alcohol consumption.
