# Supplementary material for: Association between the dietary index for gut microbiota and metabolic syndrome: the mediating role of the dietary inflammatory index
Source: Front Nutr. 2025 Jul 21;12:1617287. doi: 10.3389/fnut.2025.1617287 (PMC12318735; doi:10.3389/fnut.2025.1617287)
Supplement: Supplementary file 4 [file Table_4.docx]

**Supplementary Table 4.** The correlation between DI-GM and the prevalence of metabolic syndrome in the study participants, further adjusted for macronutrient intake and physical activity.

| Variable | Model1 |  | Model2 |  | Model3 |  |
| --- | --- | --- | --- | --- | --- | --- |
|  | OR(95%CI) | P | OR(95%CI) | P | OR(95%CI) | P |
| MetS |  |  |  |  |  |  |
| DI-GM Continous | 0.93(0.90,0.97) | <0.001 | 0.89(0.86,0.92) | <0.001 | 0.91(0.87,0.94) | <0.001 |
| Quartile 1 | Ref. |  | Ref. |  | Ref. |  |
| Quartile 2 | 0.94(0.81,1.09) | 0.4 | 0.90(0.77,1.04) | 0.2 | 0.91(0.78,1.07) | 0.3 |
| Quartile 3 | 0.76(0.64,0.89) | 0.001 | 0.67(0.56,0.79) | <0.001 | 0.70(0.59,0.83) | <0.001 |
| Quartile 4 | 0.76(0.68,0.89) | <0.001 | 0.61(0.51,0.71) | <0.001 | 0.65(0.55,0.77) | <0.001 |
| Elevated FPG |  |  |  |  |  |  |
| DI-GM Continous | 0.95(0.91,0.98) | 0.002 | 0.92(0.88,0.95) | <0.001 | 0.93(0.90,0.97) | <0.001 |
| Quartile 1 | Ref. |  | Ref. |  | Ref. |  |
| Quartile 2 | 0.89(0.76,1.04) | 0.2 | 0.89(0.76,1.05) | 0.2 | 0.90(0.77,1.06) | 0.2 |
| Quartile 3 | 0.86(0.73,1.00) | 0.053 | 0.82(0.69,0.98) | 0.026 | 0.84(0.71,1.01) | 0.062 |
| Quartile 4 | 0.78(0.66,0.92) | 0.003 | 0.71(0.60,0.83) | <0.001 | 0.72(0.60,0.86) | <0.001 |
| Low HDL-C |  |  |  |  |  |  |
| DI-GM Continous | 0.94(0.91,0.96) | <0.001 | 0.93(0.90,0.96) | <0.001 | 0.93(0.91,0.96) | <0.001 |
| Quartile 1 | Ref. |  | Ref. |  | Ref. |  |
| Quartile 2 | 0.98(0.84,1.14) | 0.8 | 0.97(0.83,1.13) | 0.7 | 0.96(0.82,1.13) | 0.6 |
| Quartile 3 | 0.92(0.79,1.07) | 0.3 | 0.92(0.79,1.06) | 0.3 | 0.92(0.79,1.06) | 0.2 |
| Quartile 4 | 0.72(0.63,0.83) | <0.001 | 0.71(0.62,0.82) | <0.001 | 0.71(0.62,0.82) | <0.001 |
| Elevated WC |  |  |  |  |  |  |
| DI-GM Continous | 0.94(0.92,0.97) | <0.001 | 0.89(0.87,0.92) | <0.001 | 0.93(0.90,0.96) | <0.001 |
| Quartile 1 | Ref. |  | Ref. |  | Ref. |  |
| Quartile 2 | 1.02(0.90,1.16) | 0.8 | 0.96(0.84,1.08) | 0.5 | 1.01(0.88,1.16) | 0.8 |
| Quartile 3 | 0.84(0.74,0.96) | 0.01 | 0.75(0.65,0.86) | <0.001 | 0.82(0.71,0.95) | 0.008 |
| Quartile 4 | 0.79(0.69,0.90) | <0.001 | 0.63(0.54,0.74) | <0.001 | 0.72(0.61,0.85) | <0.001 |
| Elevated BP |  |  |  |  |  |  |
| DI-GM Continous | 0.99(0.96,1.02) | 0.7 | 0.92(0.89,0.96) | <0.001 | 0.94(0.91,0.98) | 0.001 |
| Quartile 1 | Ref. |  | Ref. |  | Ref. |  |
| Quartile 2 | 0.94(0.83,1.08) | 0.4 | 0.89(0.77,1.02) | 0.1 | 0.86(0.74,1.01) | 0.06 |
| Quartile 3 | 0.92(0.78,1.08) | 0.3 | 0.77(0.64,0.93) | 0.008 | 0.79(0.63,0.98) | 0.031 |
| Quartile 4 | 1.00(0.87,1.14) | >0.9 | 0.72(0.62,0.85) | <0.001 | 0.75(0.64,0.89) | 0.001 |
| Elevated TG |  |  |  |  |  |  |
| Quartile 1 | 1.20(0.97,1.48) | 0.1 | 1.25(1.01,1.55) | 0.045 | 1.24(0.99,1.54) | 0.06 |
| Quartile 2 | 1.30(1.03,1.63) | 0.027 | 1.33(1.06,1.69) | 0.017 | 1.32(1.04,1.67) | 0.023 |
| Quartile 3 | Ref. |  | Ref. |  | Ref. |  |
| Quartile 4 | 0.94(0.76,1.15) | 0.5 | 0.91(0.74,1.12) | 0.4 | 0.90(0.73,1.10) | 0.3 |

**Notes:** Data are presented as weighted odds ratios (OR) with 95% confidence intervals (CI). Model 1 is the crude model. Model 2 is adjusted for gender, age, race. Model 3 is further adjusted for education, marital relations, PIR, smoking, medication history, energy ,alcohol consumption, Carbohydrates, Protein, Total fats and Physical activity.
